# Anatomical Study of the Lingual Nerve and Inferior Alveolar Nerve in the Pterygomandibular Space: Complications of the Inferior Alveolar Nerve Block

**DOI:** 10.7759/cureus.3109

**Published:** 2018-08-06

**Authors:** Joe Iwanaga, Paul J Choi, Marc Vetter, Mayank Patel, Shogo Kikuta, Rod J Oskouian, R. Shane Tubbs

**Affiliations:** 1 Medical Education and Simulation, Seattle Science Foundation, Seattle, USA; 2 Surgery, Seattle Science Foundation, Seattle, USA; 3 Seattle Science Foundation, Seattle, USA; 4 Clinical Anatomy Research, Seattle Science Foundation, Seattle, USA; 5 Dental and Oral Medical Center, Kurume University School of Medicine, Kurume, JPN; 6 Swedish Neuroscience Institute, Seattle, USA; 7 Neurosurgery, Seattle Science Foundation, Seattle, USA

**Keywords:** anatomy, cadaver, dissection, inferior alveolar nerve, lingual nerve, nerve block

## Abstract

The inferior alveolar nerve block (IANB) procedure delivers anesthetics to the pterygomandibular space through which the lingual nerve (LN) and inferior alveolar nerve (IAN) travel. Injury to the LN has been reported more often than injury to the IAN. However, the number of anatomical studies of LN injury is limited. We aimed to establish evidence by investigating LN and IAN anatomy at the level of the mandibular foramen (MF). Forty-four sides from 22 Caucasian cadaveric heads (16 fresh-frozen and six formalin-fixed cadavers) were used in this study. The LN and IAN were laterally dissected, and the diameter and the distance between the two nerves were measured at the level of the MF. The mean diameters of the LN and IAN were 2.57 mm and 2.53 mm in fresh-frozen specimens and 2.97 mm and 2.93 mm in formalin-fixed specimens, respectively. The mean diameters of the LN and IAN in all the specimens were 2.65 mm and 2.64 mm. The distance between the posterior edge of the LN and anterior edge of the IAN at the level of the MF ranged from 1.62 to 8.36 mm with a mean of 5.33 ± 1.88 mm. These findings could elucidate the risk of LN injury during the IANB procedure.

## Introduction

Inferior alveolar nerve block (IANB) is one of the most common nerve block procedures in dentistry. It delivers anesthetics to the pterygomandibular space through which the lingual nerve (LN) and the inferior alveolar nerve (IAN) travel. To achieve a successful blockade, the anatomy of these two nerves needs to be understood. The development of cone-beam computed tomography (CBCT) has contributed to the body of observations of the mandibular foramen (MF), its related canal, and the course of the IAN inside the mandible [1]. However, a few previous anatomical studies have described the course of the nerves outside the mandible in detail. Behnia et al. [2] investigated 669 LNs in fresh cadavers and revealed the relationship between the LN and the lingual cortical plate of the mandible at the wisdom tooth. Iwanaga [3] demonstrated the different courses of the LN in the pterygomandibular space, retromolar area, and the oral floor depending on tongue movement.

IANB could injure the LN and IAN by direct needle insertion, chemical trauma, and hematoma [4-7]. Although the actual incidence of LN and IAN injury due to the injection itself has not been reported in the English literature [8], the incidence of permanent injury to the LN, IAN, or both is between 1 in 26,762 and 1 in 160,571 IANBs [9]. The IANB procedure more frequently injures the LN than the IAN [9-11]. One study offered a possible explanation by counting the number of fascicles histologically [12]; there were fewer LN fascicles in the area of needle insertion. Also, the LN is more likely to be exposed to the needle than the IAN at the injection site [8,12].

Lingual nerve injury by direct needle insertion could result from its anatomical features. Our aim in this study was to establish anatomical evidence by investigating LN anatomy at the level of the MF where the IANB is used.

## Materials and methods

The anatomical quality assurance (AQUA) checklist was used for this study [13-14]. Forty-four sides from 22 Caucasian cadaveric heads (16 fresh-frozen and six formalin-fixed) were used. The specimens were derived from 11 males and 11 females, and the age at death ranged from 56 to 99 years with a mean of 76.6 ± 11.9 years. Mandibular tooth status was recorded as “dentulous” when the ipsilateral side of the mandible had one or more teeth or “edentulous” when it had none.

**Figure 1:**
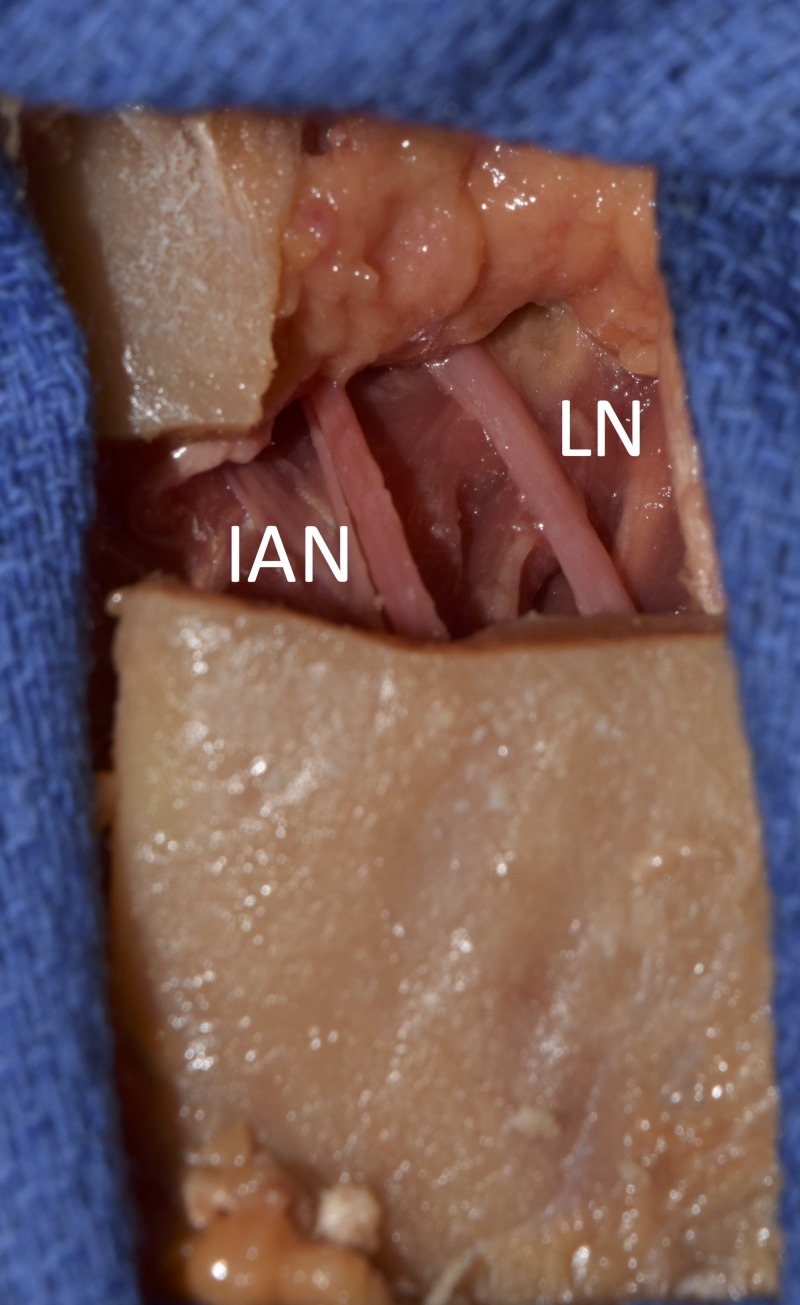
Exposure of the right lingual nerve (LN) and inferior alveolar nerve (IAN) in a fresh cadaver

All the heads were dissected with the mouth closed. On all sides, the skin of the lateral face was removed first, and then the masseter muscle and the zygomatic arch were removed. The periosteum attached to the medial aspect of the mandibular ramus was carefully detached by a periosteum elevator from superiorly to inferiorly to confirm the position of the MF and the inferior alveolar neurovascular bundles. Next, the upper portion of the mandibular ramus including the coronoid process with the insertion of the temporalis was cut horizontally just above the MF, so as not to damage the IAN, and removed (Figure 1).

The LN and IAN were dissected and their diameters and the distance between the posterior edge of the LN and the anterior edge of the IAN were measured at the level of the MF.

One clinical anatomist and one oral and maxillofacial surgeon performed all the dissections and measurements. All of the measurements were made with a microcaliper (Mitutoyo, Kanagawa, Japan). This study protocol did not require the ethics committees’ approval in our institutions, and the work was performed in accordance with the requirements of the Declaration of Helsinki (64th WMA General Assembly, Fortaleza, Brazil, October 2013).

## Results

On all 44 sides, the LN was identified anterior to the IAN. Twenty-two sides were dentulous and 22 edentulous. The diameter of the LN ranged from 1.53 to 3.98 mm with a mean of 2.65 ± 0.49 mm. The diameter of the IAN ranged from 1.77 to 3.89 mm with a mean of 2.64 ± 0.39 mm. There were no significant differences in diameter between the LN and IAN (p>0.05) (Table 1).

**Table 1:** Diameter of lingual and inferior alveolar nerves

	Range (mm)	Mean (mm)
Lingual nerve	1.53 to 3.98	2.65±0.49
Inferior alveolar nerve	1.77 to 3.89	2.64±0.39

The mean diameters of the LN in fresh-frozen and formalin-fixed cadavers were 2.57 ± 0.44 and 2.97 ± 0.48 mm, respectively. The mean diameters of the IAN in fresh-frozen and formalin-fixed cadavers were 2.53 ± 0.32 and 2.93 ± 0.40 mm, respectively. The diameters of both the LN and IAN differed significantly between the fresh and formalin-fixed cadavers (p<0.05). The distance between the posterior edge of the LN and the anterior edge of the IAN at the level of the MF ranged from 1.62 to 8.36 mm with a mean of 5.33 ± 1.88 mm. There were no significant differences in diameter or distance with respect to tooth status, sides, or sex (p>0.05). No previous injury to the nearby IAN or LN was observed on any cadaveric side.

## Discussion

**Figure 2:**
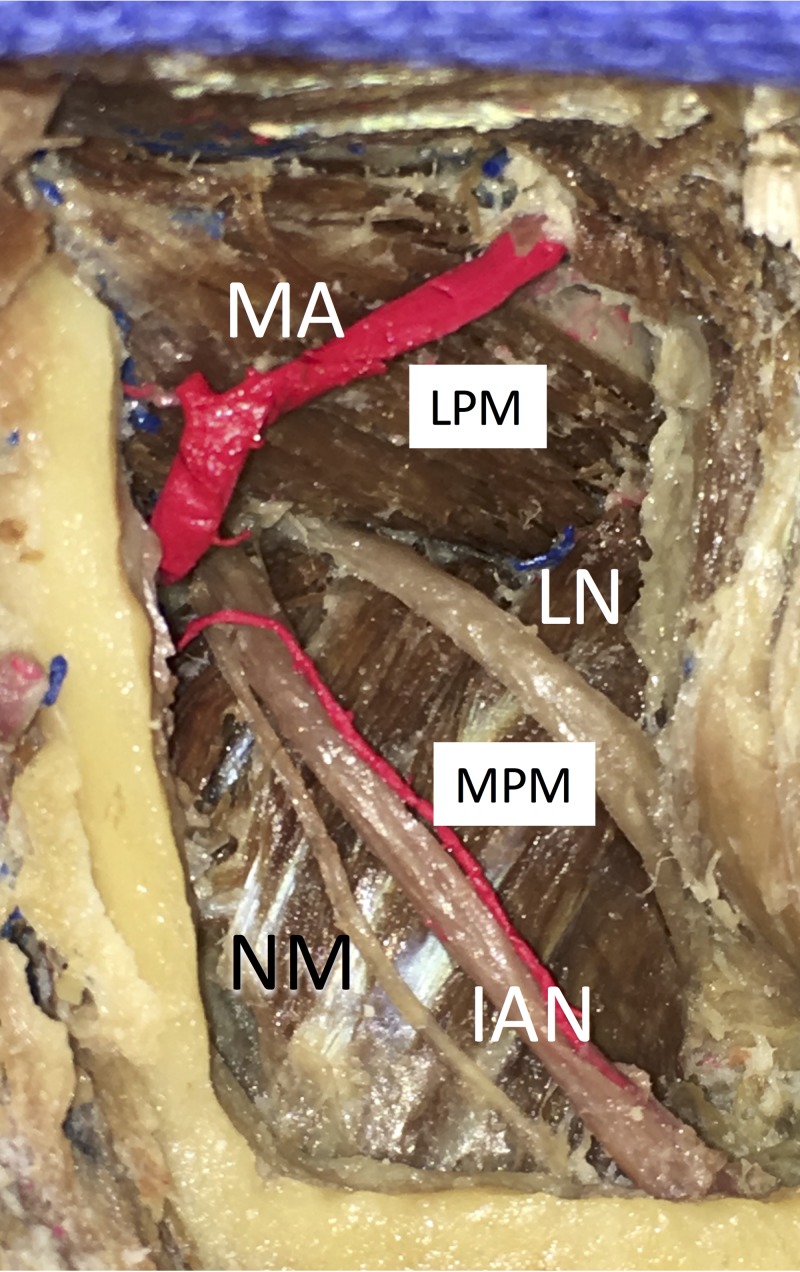
Lateral view of the right pterygomandibular space in a formalin-fixed cadaver. Note that the lingual and inferior alveolar nerves are compressed by the medial pterygoid muscle IAN: inferior alveolar nerve, LN: lingual nerve, LPM: lateral pterygoid muscle, MA: maxillary artery, MPA: medial pterygoid muscle, NM: nerve to mylohyoid

The anatomy of the LN is critical for considering the complications of IANB and wisdom tooth extraction. Its course in the retromolar area and oral floor, and its communicating branches with the hypoglossal nerve are well known [2,8,15]. The diameter of the LN has been given as 1.84 mm in a cadaveric study [16] and 2.54 mm in an imaging study [17] at the position of the wisdom tooth. Thus, it seems we have enough data to prevent injury to the LN injury during an IANB and wisdom tooth extraction. However, only one previous study investigated the diameters of the two nerves at the level of the needle injection [18], although it is empirically known that they are approximately the same at this site. As our results showed, the mean diameters of the LN and IAN differed significantly between the fresh-frozen (LN: 2.57 mm, IAN: 2.53 mm) and formalin-fixed (LN: 2.97 mm, IAN: 2.93 mm) specimens (p<0.05), although the mean diameters of both the LN and IAN in all the specimens were almost equal (LN: 2.65 mm, IAN: 2.64 mm). These differences between fresh-frozen and formalin-fixed specimens could be due to the compression of the nerves by the medial pterygoid muscle and the ramus of the mandible in formalin-fixed cadavers (Figure 2).

In fresh specimens, where the nerves were measured, the medial pterygoid muscles were not usually compressing them. According to Morris et al. [18] the mean diameter of the LN in formalin-fixed cadavers at the horizontal plane of needle placement was 3.42 ± 0.38 mm. The present study also demonstrated that the LN was located anterior to the IAN in all sides, and the mean distance between the two nerves at the level of the MF was 5.33 ± 1.88 mm. The anteroposterior distance between the nerves was also measured in this study, and the close relationship between the LN and IAN and their similarity of diameter were confirmed. These results support the view that direct needle insertion into the LN is more common than into the IAN. As the course of the LN can change depending on the position of the tongue or mouth opening [3], a more dynamic future study with tongue or mouth movement is required.

## Conclusions

The results of this study showed that the mean diameters of the LN and IAN had a statistically significant difference between fresh-frozen and formalin-fixed specimens. Moreover, the LN was located anterior to the IAN on all sides. The close relationship between the LN and IAN and their similarity of diameter were demonstrated.
